# Global Thrombosis Test: Occlusion Is Attributable to Shear-Induced Platelet Thrombus Formation

**DOI:** 10.1055/a-1704-1022

**Published:** 2021-11-23

**Authors:** Diana A. Gorog, Junichiro Yamamoto

**Affiliations:** 1 Faculty of Medicine, National Heart and Lung Institute, Imperial College, London, United Kingdom; 2 Clinical Pharmaceutical and Biological Science, School of Life and Medical Science, University of Hertfordshire, United Kingdom; 3 Faculty of Nutrition, Kobe Gakuin University, Kobe, Japan

We read the paper by Clavería and co-workers with interest. 1 We strongly believe that the conclusions of their paper, suggesting that occlusion (occlusion time (OT)) in the global thrombosis test (GTT) is due to coagulation, rather than shear-induced platelet thrombus formation, is wrong and the evidence and arguments they present are fundamentally flawed.

The authors have made major errors both in the approach to their experiments and in their interpretations of their results. The evidence which they demonstrate, shows that occlusion in the GTT is, in fact, caused by high shear-induced platelet thrombus formation, the complete opposite to their interpretation of their results. We set out below the evidence for that.

## Evidence that Occlusion in the GTT Is Caused by High Shear-Induced Platelet Thrombus Formation

### Histology

In the paper, Clavería et al state that “Histology of clot formed and retracted long after occlusion showed predominantly red cells with plenty of fibrin fibers, but platelets could not be seen anywhere, neither between the two ceramic balls, nor at the place, in the gaps where they should have been, if they were the reason for occlusion.”

Our first concern is the extraction of apparently only the head of the thrombus between the bead and the wall of the tubing. Firstly, the authors claim to have extracted a minuscule thrombus from a 38-µm width gap between the inner surface of the plastic tube and the ceramic bead—this is some feat, especially when they stated that it was not visible to the naked eye at the time of the occlusion. There is no description of methodology. On a practical level, it is impossible to just extract this “thrombus” from a 38-µm gap and the authors would have to remove the whole clot that is much larger and therefore contains the “red tail” of the thrombus.

More worryingly, the authors do not “fix” the extracted thrombus in glutaraldehyde or other fixative and do not arrest the thrombus formation/coagulation process. Thus, the time it takes from the very fiddly attempt to remove the thrombus to analysis, clotting will be occurring in the extracted blood sample (since it is not anticoagulated). The authors are therefore presenting an analysis of a clotted blood sample, not the platelet thrombus.

Arrest of flow (OT) in the GTT is due to thrombotic occlusion of small gaps adjacent to the smaller (3 mm) bead. In their interpretation of the histology in Fig. 2, Clavería et al actually observe that in the gaps by the smaller bead “some light blue tinge appearing close to all artificial surfaces and dark blue stain (high platelet concentration) could be observed at the extensions (presumably the gaps) at the lower 3-mm bead.” This is, by all definitions, clear proof of high platelet concentration in the area of interest. Yet the authors ignore their own findings and claim the opposite, namely that “we could not distinguish from the histology whether flow occlusion was predominantly from clot in the gaps or from the large clot forming between the beads.”

In a prior publication, using a more careful technique, at 90 seconds from the start of measurement, the two beads and blood were removed from the test tube by aspiration; the inner wall of the plastic tube was cut into segments and fixed. 2 Examination by scanning electron microscopy showed an absence of any platelet aggregates between the two ball bearings. However, close to the gaps adjacent to the position of the lower ball, large platelet aggregates with extending pseudopods were frequently seen. These platelet aggregates were attached to the plastic surface by fine fibrin strands. More recently in patients with ST-elevation myocardial infarction (STEMI), we carefully extracted the whole thrombus, immediately washed in Na-cacodylate buffer and fixed with 2.5% glutaraldehyde, before point-critical dehydration with ethanol and hexamethyldisilazane. Scanning electron microscopy showed a high density of platelet-fibrin thrombus; red cells were not seen in the extracted (and importantly, fixed) head of the thrombus. 3

### Effect of Heparin on OT

The authors argue that because heparin was shown to significantly prolong the OT, then the “thrombus” formed must be similar to a venous clot, rather than an arterial thrombus. They state that “heparin strongly inhibits coagulation and the formation of fibrin so that we might expect increases in OT if clots are fibrin rich. This strong effect by heparin suggests that the main cause for occlusion in the GTT is coagulation.”

This conclusion is wrong on multiple levels. Firstly, in addition to its well-known anticoagulant effect, heparin is known to exert an inhibitory effect on platelet aggregation induced by various platelet agonists 4 and that heparin neutralizes the inhibitory effect of prostacyclin (PGI2) on platelet aggregation. 5 Under high shear conditions, heparin has been shown to strongly inhibit platelet aggregation in 91.4% of patients with cardiovascular disease. 6 Other techniques, even with lower shear, have shown an inhibitory effect of heparin on platelet activation and aggregation. Using multiple electrode aggregometry, low molecular weight heparin was shown to significantly reduce platelet activation, 7 and using the PFA 100, collagen/adenosine-5'-diphosphate (ADP) closure times were significantly prolonged by heparin administration, and reversed with protamine. 8 9 Using the Clot Signature Analyzer assessing collagen-induced thrombus formation under moderate shear and high shear platelet activation, heparin inhibited both high shear collagen-independent and moderate shear collagen-dependent platelet activation. 10

Secondly, the authors appear not to appreciate that fibrin is an essential constituent of arterial thrombi (not only venous clots), with arterial thrombi predominantly composed of platelet aggregates held together by a fibrin mesh. 11 12 The role of fibrin in platelet thrombi is well established from animal studies and post-mortem studies. 13 14 In vivo, extraction of intracoronary thrombi from patients with STEMI showed that thrombi were mainly composed of fibrin (55.9 ± 18%) with platelets (16.8 ± 18%), erythrocytes (11.5 ± 9%), cholesterol crystals (5.2 ± 8.4%), and leukocytes (1.3 ± 2.0%). 15 Characterization of coronary thrombi from patients with acute coronary syndromes (ACS) using dye-staining angioscopy showed fibrin-rich thrombus in 60% of non-ST elevation ACS (NSTE-ACS) and in 29% STEMI patients. 16 By histology, fibrin dominant thrombus was observed in 71% of NSTE-ACS patients and in 28% of STEMI patients. In contrast to the generally believed concept that white coronary thrombi are composed of platelet aggregates, white thrombi rich in fibrin were frequently observed. 17 18

Thus, in addition to platelet thrombi, fibrin thrombi play an important role in the genesis of ACS. Because fibrin cannot be imaged by other modalities, dye-staining angioscopy can help clarification of roles of fibrin in the mechanism of coronary thrombus formation. 16 An elegant recent review on the composition of thrombus in patients with cardiovascular disease demonstrates that thrombi are heterogenous in composition, but overall, thrombi obtained from patients with ACS are composed of mainly fibrin and other components, including platelets, red blood cells, leukocytes, and cholesterol crystals. In contrast thrombi from patients with venous thromboembolism contain mainly red blood cells and fibrin with some platelets and leukocytes. 14

### Effect of t-PA on OT

The authors use their finding that addition of t-PA significantly shortened the lysis time in the GTT as evidence that the “thrombotic occlusion in GTT (is) strongly dominated by coagulation.” t-PA is the most widely used thrombolytic agent in the world and works by lysing *arterial* thrombi and achieving reperfusion, thereby reducing infarct size. Amongst patients with occlusive arterial thrombosis, namely patients with STEMI, administration of t-PA within 90 minutes restores vessel patency in 75% of patients 19 and similar patency rates are achieved when t-PA is given to patients with acute ischemic stroke, with t-PA frequently used for these indications. 20 In contrast, data showing the effectiveness of t-PA in patients with deep venous thrombosis (DVT) is lacking, and there is no role of t-PA in routine management of DVT. 21 In an early study with the GTT, streptokinase and t-PA greatly shortened the lysis times, while neither of them had any effect on OT. 2

Therefore, the fact that the authors show that t-PA significantly shortens lysis time supports the notion that the thrombus formed in the GTT is more akin to an arterial, than a venous thrombus.

### Unique modification of GTT (mGTT)

Bizarrely, Clavería et al sought to “modify and improve the GTT” by removing one of the two beads in the GTT, leaving only the larger bead and coating it with collagen. The authors claim that with this modification they “avoided the low shear zone between the beads, where the clots formed in the GTT.” They are correct and in fact, with their “modification,” they render the GTT no longer pathologically relevant to arterial thrombosis, for three reasons. First, in this modification, as blood flows past the sole bead now coated with collagen, relatively stable platelet aggregates are formed in the proximity of the gaps, causing much more rapid occlusion and arrest of flow, as the authors show. However, the nature of the initial stimulus for occlusion is no longer the pathologically relevant high shear, but rather the contact pathway of coagulation. Second, in a landmark paper, Jackson et al 22 elucidated the mechanism of platelet thrombus formation by high shear and analysis of blood flow dynamics revealed that whilst platelet activation occurred specifically at the apex of the stenosis, subsequent platelet aggregates formed preferentially in the downstream expansion zone. This area is replicated adjacent to the second bead in the GTT, and is exactly what Clavería and co-authors have removed with their modification, by which they render the GTT unphysiological. Finally, as the authors also state, the modification prevents the possible measurement of endogenous thrombolysis, a unique feature of GTT test.

### Ignoring Evidence on the Effect of Antiplatelet Drugs on OT

The authors suggest that the “clot” formed in the GTT is akin to a venous thrombus. However, the importance of platelets in the occlusive thrombus formation in the GTT was demonstrated by our group, showing that P2Y _12_ inhibitors significantly prolong OT, compared with baseline (pre-P2Y _12_ inhibitor treatment), with the magnitude of OT prolongation being directly proportional to the potency of the P2Y _12_ inhibitor used. 23

### Duration of OT

We believe the authors’ claim that OT “in GTT ….occludes in approximately 8 minutes (is) consistent with the kinetics on the coagulation cascade,” is inaccurate. Clotting time of non-anticoagulated whole blood in plastic tubes takes much longer and reported to take an average of 13 minutes under static conditions. 24 In the GTT, since blood is flowing, dynamic coagulation takes much longer than in a stagnant environment. 25

Under dynamic flow conditions, clotting times of 16.5 minutes, 2 18.8 minutes, 26 and 22.2 minutes have been reported in the literature. 27 In an elegant experiment Yamamoto et al 2 measured the dynamic clotting time in the GTT in two settings: (1) the lower bead was removed, and (2) both beads were removed. The flow rate was kept constant during the measurements. When the lower bead was removed, the dynamic clotting time was 16.5 minutes. When both the beads were removed, the dynamic clotting time could not be measured, as it did not occur until the blood ran out at 22 minutes. The observation, that when a single bead was in place and shear-induced platelet activation occurred, the observed clotting time was 16.5 minutes in comparison to when both beads were removed and the stimulus for platelet activation was absent, the clotting time was >22 minutes, strongly supports the role of shear-activated platelets contributing to the dynamic coagulation of blood. Published data from our group on two different sets of 100 healthy volunteers show that OT was achieved in 4.7 ± 1.2 minutes, in other words, in less than one-third of the time that it takes to achieve clotting under static conditions, 28 29 and also less than one-third of the time it takes to achieve clotting under dynamic flow conditions, 2 26 implying that OT is due to platelet thrombus formation rather than coagulation.

Additionally, the finding that OT in “hypershear” mode is almost half the time taken to achieve OT in GTT-2 mode is a further evidence that this technique reflects shear-induced occlusion, the speed of which varies with the extent of shear.

### Variation in OT between Different “Bead Materials” Table 2—the Difference Is Actually due to Different Populations Studied

Clavería et al use Table 2 in their paper to demonstrate differing OTs measured in different papers and state that this is due to the differences in bead characteristics in various iterations of the GTT. They seem to have missed the point that these are different patient cohorts and hence they will of course be different. In addition to known variation in platelet function/thrombotic status by age and cardiovascular risk factor status, we and others have also reported ethnic variation in OT and LT, between East Asians and White Europeans, including with the GTT. 29 To specifically exemplify some of the differences in the populations studied in the different papers cited in Table 2, one reference measured OT in healthy young Japanese volunteers (average age 37.9 years), 30 another in Japanese volunteers with an average age 69 years, 31 another assessed predominantly Caucasian volunteers (average age 37 years), 28 another assessed Japanese habitual smokers, 32 another assessed predominantly Caucasian patients with advanced cardiovascular disease taking both aspirin and clopidogrel at the time of sampling (mean age 60 years). 33

Furthermore, looking specifically at the studies that appear to use the same populations, those assessing OT in healthy Japanese populations 31 32 34 35 report similar OTs, regardless of whether stainless steel balls [OT 464.5 35 and OT 495 32 ] or ceramic balls [OT 481 34 and 524.9 31 ] were used.

Lastly in Table 2, the authors cite two different values for OT from the reference by Ikarugi et al, 36 but fail to point out that one was obtained in a population of elderly men who were habitual smokers and the other obtained from healthy young volunteers.

Therefore, we show that differences in OT are not simply attributable to the beads or the instrument, these are different populations being tested.

### Importance of Collagen

The authors' claim that “shear-induced thrombi form in the presence of three factors: (1) Von Willebrand factor (vWF) and platelets in whole blood; (2) high shear rates; and (3) fibrillar collagen surface”—is inaccurate in artificial systems. Naturally, thrombotic occlusion in a sizeable artery can occur only if the thrombus is firmly attached to the vessel wall due to endothelial disruption and exposure of subendothelial collagen-rich material. However, small, nonocclusive thrombi can form and embolize, causing downstream occlusion of distal small vessels.

Paul Didisheim first demonstrated that a hemostatic plug can form in a plastic tubing that is not coated with collagen. 37 In the Chandler loop, one of the best recognized laboratory techniques, to this day, to evaluate thrombus formation initiated by shear forces, thrombus is formed in a plastic tubing without collagen. 38 39 In yet another system in which shear-activated platelet activation on the surface of uncoated glass beads caused a large platelet aggregate formation resulting in arrest of flow, collagen was not required. 40 Whilst it is true that in the absence of fibrillar collagen, the interaction of platelets with the plastic surface is weak and results in only microthrombi which never cause full luminal occlusion of an ordinary plastic tube, the large platelet aggregates formed in the low shear zone downstream of the stenosis/shear-activation site can propagate or embolize causing downstream occlusion of smaller distal vessels. This situation is mimicked by the GTT, such that collagen coating of the beads is not required.

## Evidence of Inadequate Sample Handing by Clavería et al

Since blood is not anticoagulated, it is essential to introduce the blood sample into the GTT instrument within 15 seconds of blood draw. Yet the authors provide evidence that they were introducing an already partly coagulated sample into the instrument in their paper in Fig. 11a which they showcase as an example of a “normal” curve, explicitly stating in the legend “In (a) the stored values increase smoothly and monotonically, as it is expected from the measurements.”

**Fig. 1:**
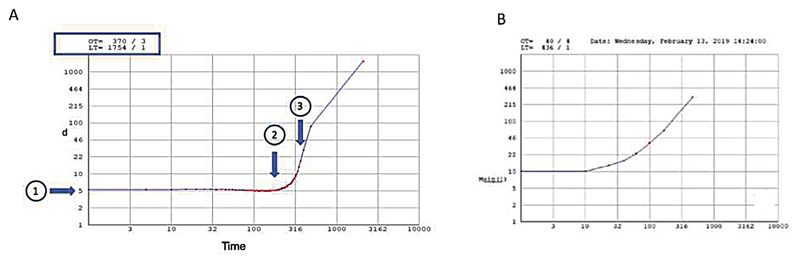
( **A** ) Example of a normal, physiological readout from the GTT from our group. The time between consecutive drops (d) is measured at 5 at the start of measurement (arrow 1), with gradual reduction in flow shown by increasing (d) ( *arrow 2* ) and eventual occlusion time ( *arrow 3* and also shown as readout on top left-hand corner of graph as OT 370). ( **B** ) Reproduction of readout of “normal” curve from Clavería et al showing at the start of the measurement, (d) is already 10, with rapid increase in (d) at 10 seconds (on x-axis) with resultant OT 40 seconds, which represents an erroneous reading—in contrast to data from a number of prior studies all showing average OT in normal volunteers between 350–490 seconds. 28 29 34 35

It is concerning, that the authors do not recognize that this is a very abnormal curve, which is shown in three ways in their graph. We demonstrate the problem below, comparing our normal curve, with the erroneously labelled “normal” curve in Clavería's paper ( Fig. 1 ).

At the start of measurement, the time difference between the two consecutive blood droplets (d) relates to the viscosity of blood. In our experience using the GTT tubes with ceramic beads, at the start of measurement, (d) should be 5 ± 2 seconds (shown on the y-axis).

We demonstrate an example of a normal readout from our group ( Fig. 1A ), showing *d* = 5 at the start of measurement (arrow 1), with gradual reduction in flow shown by increasing (d) (arrow 2) and eventual occlusion time (arrow 3 and also shown as readout on top left-hand corner of graph as OT 370). In contrast, in Fig. 1B (reproduced from Fig. 11a from Clavería et al), at the start of the measurement, already *d* = 10 demonstrating increased viscosity and longer time between drops than normal. At 10 seconds (on x-axis) we see (d) rapidly increase, showing reduction in flow, due to increased viscosity, most probably caused by activated coagulation during or after blood sampling and before the sample was introduced into the instrument. Finally, as can be seen at the top of the graph, the measured OT is 40 seconds. This is wholly unphysiological and reflects a clotted sample.

Pertinent to this, we and others have shown that the inter-assay and intra-assay coefficients of variation were 8 and 9% for OT and 10 and 10% for LT, respectively. 3

## Reported Technical Problems

The authors claim that there is variation in gap size in the GTT consumable test tube. As clinicians and researchers, we are not in the habit of disassembling research or clinical tools to check for consistency with micro-CT scanning. However, the coefficient of variation (cv) with this technique in both our centers is very good and performs similarly (or better) than cv of other platelet function tests. The authors notably state that “As the beads are in a conical section, small changes in angulation of the tube may cause distortions in the visualization of the gaps and in the wall thickness. The ambiguity in gap size made calculation of the true shear rates difficult.” As the authors point out, this is an unreliable way to assess gap sizes and is fraught with error.

The authors claim that the size of blood drops showed great variation when it was measured in several test tubes outside the instrument. The size of each blood drop is determined by the viscosity of blood, which will also impact the surface tension, and viscosity is also influenced by ambient temperature when the authors removed the plastic tube from the instrument. In contrast to the blood tested in the GTT which is maintained at 37°C, the authors conducted their experiments *outside* of the GTT, at ambient room temperature. It was shown that reducing blood temperature from 36.5° to 22°C, increases blood viscosity by 26.13%, leading to a 20.72% decrease in blood flow rate. 41 Therefore, the most likely explanation for the variation in droplet size outside of the GTT was the decreasing temperature of blood, leading to increased viscosity and reduced flow.

## Problems with Graphs

To illustrate the problems with Data Readouts, in Fig.11 the authors demonstrated the differences between data displayed by the instrument display panel and the graph formed from the saved data on SD card. Three graphs were selected as examples.

Example (1) the authors show this graph to exemplify a “normal” tracing, while it is far from normal. The OT of 40 seconds indicates a profound hypercoagulation, the most likely cause is improper blood sampling or introducing an already partly coagulated sample into the instrument.

Example (2) shows a test where the blood started to flow into the container part of the test tube only after 361 seconds from the transfer of blood sample into the test tube. Under normal circumstances, the first drop of blood should be indicated on the instrument display within 15 seconds of the transfer of blood into the tube.

Example (3) demonstrates two graphs superposed on each other with lines of interference caused by the computer display. This happens when a measurement (file) with a memory number is saved when another measurement with an identical memory number was already saved in the computer previously. This suggests that the authors were not using the latest version of GTT-Draw software, which would have prevented such errors.

## General Points

Quite some time ago the mechanisms of arterial and venous thrombosis were regarded as totally separate entities. In coagulation, clotting factors and thrombin, while in arterial thrombosis, platelets were regarded as major players. Today the accepted model of arterial thrombosis is one of a cell-based control of coagulation, with platelets not only acting as a scaffold for assembly of coagulation factors and the generation of thrombin, but also supporting fibrin formation, and regulation of fibrin clot retraction. 42

Relevant to high shear-induced platelet aggregation, there is a delay in aggregation response to shear rates approximately 10,000 s ^−1^ . Following platelet activation by high shear, it takes time to form larger aggregates since this requires the release of soluble agonists from alpha and dense granules and surface exposure of phosphatidylserine in shear-activated platelets, to generate thrombin and enable fibrin stabilization of the formed aggregates.

Shear levels variably affect the platelet–vWF interaction. 43 Under a threshold of 800 s ^−1^ , there is no platelet–vWF interaction; the dominating platelet-activating stimuli being the exposed subendothelium or collagen and/or soluble agonists released from platelet granules. In the shear range of 1,000 to 10,000 s ^−1^ , such as those typically found in the arterial microcirculation or in regions of moderate arterial stenosis, the platelet–vWF interaction-induced aggregation becomes increasingly significant. At pathological shear rates >10,000 s ^−1^ , platelet–vWF interaction is the dominant initiator of platelet aggregation and thrombus formation. However, thrombi do not form even at a steady high shear rates of 20,000 s ^−1^ , because it is the shear gradient which plays the main role in thrombus formation in a stenotic artery. 22 At a relatively low input shear rate of 1,800 s ^−1^ upstream of the stenosis, a shear rate of 20,000 s ^−1^ can be generated at the apex of stenosis with rapid deceleration to about 800 s ^−1^ in the post-stenosis area, where accelerated platelet aggregation occurs.

By exposing platelets to shear rate of around 10,000 s ^−1^ , the GTT emulates the above shear-gradient induced thrombus formation and growth, which is almost entirely based on the interaction of platelets with vWF. However, because of the very short shear-exposure time (milliseconds) while platelets pass through the very narrow gaps between the upper bearing ball and the inner surface of the plastic test tube, the efficiency of shear activation is less than it would be measured in a parallel plate flow chamber, where platelets are exposed to shear for as long as 20 to 30 seconds. In practical terms, using the GTT in “GTT-2 Basic Clinical Mode,” interaction of platelets with vWF is not the dominating initiator of platelet aggregate formation but the platelet-released soluble agonists still play a part. It is well documented, that at shear gradients of 10,000 s ^−1^ , aspirin and probably ADP antagonists are not able to inhibit platelet aggregation. However, we and others have shown, that the GTT sensitively detects the effectiveness of aspirin and P2Y _12_ inhibitor therapy. 23 This implies that in the mechanism of occlusion, both platelet activating pathways, namely platelet-vWF and soluble agonists, play a role, indicating its suitability to reflect thrombosis in both moderate and highly stenosed vessels.

At much higher shear rates (>20,000 s ^−1^ ), vWF can induce activation-independent platelet aggregation. Consequently, the time between exposure of platelets to high shear until occlusion by platelet aggregates is greatly reduced. This mechanism is simulated when GTT-3 is used in “hypershear” mode. As in the case in vivo in the formation of an arterial thrombus, similarly in the GTT, activation of platelets and coagulation is entwined. To answer the question proposed in the title of Clavería and co-workers' paper, it is essential to look solely at the nature of activating stimulus to thrombus formation; high shear does not cause coagulation, it causes platelet activation, hence the mechanism of the occlusive thrombus.

## Conclusion

In summary, we feel the authors have performed experiments with several methodological flaws and misinterpreted much of the data they obtained. We present evidence predominantly from the authors' own data, together with our earlier published data and evidence from the literature, showing that occlusion in the GTT occurs due to shear-induced platelet aggregation.
